# The ICU-venous thromboembolism score and tumor grade can predict inhospital venous thromboembolism occurrence in critical patients with tumors

**DOI:** 10.1186/s12957-022-02705-z

**Published:** 2022-09-05

**Authors:** Ruqi Mei, Guodong Wang, Renxiong Chen, Hongzhi Wang

**Affiliations:** Department of Critical Care Medicine (ICU), Key Laboratory of Carcinogenesis and Translational Research (Ministry of Education/Beijing), Peking University Cancer Hospital & Institute, Beijing, China

**Keywords:** Critically ill, Tumor patients, Pulmonary embolism, Venous thromboembolism, Deep vein thrombosis

## Abstract

**Background:**

Venous thromboembolism (VTE) is a threat to the prognosis of tumor patients, especially for critically ill patients. No uniform standard model of VTE risk for critically ill patients with tumors was formatted by now. We thus analyzed risk factors of VTE from the perspectives of patient, tumor, and treatment and assessed the predictive value of the ICU-VTE score, which consisted of six independent risk factors (central venous catheterization, 5 points; immobilization ≥ 4 days, 4 points; prior VTE, 4 points; mechanical ventilation, 2 points; lowest hemoglobin during hospitalization ≥ 90 g/L, 2 points; and baseline platelet count > 250,000/μL, 1 points).

**Methods:**

We evaluated the data of tumor patients admitted to the intensive care unit of the Peking University Cancer Hospital between November 2011 and January 2022; 560 cases who received VTE-related screening during hospitalization were chosen for this retrospective study.

**Results:**

The inhospital VTE occurrence rate in our cohort was 55.7% (312/560), with a median interval from ICU admission to VTE diagnosis of 8.0 days. After the multivariate logistic regression analysis, several factors were proved to be significantly associated with inhospital VTE: age ≥ 65 years, high tumor grade (G3–4), medical diseases, fresh frozen plasma transfusion, and anticoagulant prophylaxis. The medium-high risk group according to the ICU-VTE score was positively correlated with VTE when compared with the low-risk group (9–18 points vs. 0–8 points; *OR*, 3.13; 95% *CI*, 2.01–4.85, *P* < 0.001). The AUC of the ICU-VTE scores according to the ROC curve was 0.714 (95% *CI*, 0.67–0.75, *P* < 0.001).

**Conclusions:**

The ICU-VTE score, as well as tumor grade, might assist in the assessment of inhospital VTE risk for critically ill patients with tumors. The predictive accuracy might be improved when combining two of them; further follow-up researches are needed to confirm it.

**Supplementary Information:**

The online version contains supplementary material available at 10.1186/s12957-022-02705-z.

## Introduction

As one of the common complications in cancer patients, venous thromboembolism (VTE) became the second main cause of patient death next to tumor progression, with a 5–10% occurrence rate, 4 to 7 times higher than those without cancer, and contributed to a two- to sixfold increase in mortality as compared to matched cancer patients without VTE [1–4].

VTE was defined as deep vein thrombosis (DVT), pulmonary embolism (PE), or both. A variety of factors contribute to thrombotic risk in tumor patients, apart from patient-specific factors (age, sex, ethnicity, high body mass index, platelet count, platelet distribution width, hyperlipidemia, ABO blood type, comorbidities) [4–10] and tumor-specific factors (tumor type, anatomical location, tumor load, gene mutations, tumor stage, and pathologic grade) [1, 7, 8, 11, 12]; therapeutic measures, such as chemotherapy, the use of red cell or platelet transfusions, high-risk surgery, indwelling catheter, invasive mechanical ventilation, total parenteral nutrition (TPN), and so on, constitute the other risk factors of VTE [4, 8, 13].

Therefore, evaluating the thrombotic risk in advance appears to be particularly important for tumor patients in intensive care. As the existing International Medical Prevention Registry on Venous Thromboembolism (IMPROVE) [14] and Padua Prediction scores [15] were designed for VTE risks among hospitalized medical patients, a recent study of Viarasilpa T. developed a predictive model (the ICU-VTE score) for 37,050 critically ill patients of the Henry Ford Health System during hospitalization [5]. There is no uniform standard model of VTE risk for critically ill patients with tumors was formatted by now. The current retrospective study aimed to assess whether the ICU-VTE score, as well as patient-specific factors, tumor-specific factors, and treatment-related factors during hospitalization, were associated with inhospital VTE occurrence in critically ill tumor patients.

## Methods

### Study population

From November 2011 to January 2022, a total of 5762 patients were admitted to the intensive care unit (ICU) of the Peking University Cancer Hospital, and 560 cases were chosen for this retrospective study. We included all patients age greater than or equal to 18 years admitted to ICU for more than or equal to 24 h, who underwent emergency bedside ultrasound or spiral CT pulmonary angiography (CTPA) during hospitalization. All patients were divided into the “with VTE” group and the “without VTE” group according to ultrasound results or imaging data.

Exclusion criteria included ICU stay less than 24 h, VTE as an admission diagnosis, or when diagnosed within 24 h of hospital admission, leukemia, pregnant, or lactating.

### Data collection

We obtained patient characteristics and clinical data from the electronic medical records system of our hospital. These data were as follows: causes of ICU admissions (medical or surgical), medical diseases included infection, allergic shock, respiratory failure, and cardiac attack; age; sex; BMI; previous VTE (defined as VTE occurred before hospital admission); comorbidities of diabetes mellitus, hypertension, and main adverse cardiovascular and cerebrovascular events (MACCE); smoking history and past alcohol use; and baseline laboratory results at hospital admission including white blood cell count, hemoglobin, platelet count, prothrombin time (PT), international normalized ratio (INR), activated partial thromboplastin time (APTT), albumin, serum creatinine, bilirubin, glucose, triglycerides, cholesterol, and ABO blood group. The lowest level of hemoglobin during hospital admission was also recorded. Tumor type and location, histological grade, and tumor node metastasis staging were also recorded.

Treatments during hospitalization included surgery, blood product transfusions, mechanical ventilation, central venous catheterization (CVC), TPN, and pharmacologic prophylaxis. CVC was defined as single or double, dialysis, or tunneled catheters placed into the internal jugular, subclavian, or femoral vein, pulmonary artery catheters, and peripherally inserted central catheters. All patients received mechanical prophylaxis including graduated compression stocking (GCS) or intermittent pneumatic compression (IPC) use during ICU days before developing an inhospital VTE. In addition, anticoagulation drugs, such as low-dose unfractionated heparin, low-molecular-weight heparins (LMWH), or direct thrombin inhibitors, will be given according to the risk of bleeding during their hospitalization. Inhospital VTE was defined as acute incident DVT (either upper or lower extremity), PE, or both. We diagnosed inhospital VTE based on bedside duplex venous ultrasonography, CT venography, CTPA, or ventilation-perfusion (V/Q) nuclear imaging of the lungs once patients had clinical symptoms or changes of clinical data.

Outcome assessments included duration of mechanical ventilation, immobilization time (captured by an activity score less than 3 in the Braden Scale), ICU duration, and hospital length of stay (LOS). The ICU-VTE score which consists of six factors, CVC (5 points), immobilization ≥ 4 days (4 points), prior VTE (4 points), mechanical ventilation (2 points), lowest hemoglobin during hospitalization ≥ 90 g/L (2 points), and baseline platelet count > 250,000/μL (1 point), was used to predict thrombotic risk and was divided into three grades: low risk (0–8 points), intermediate risk (9–14 points), and high risk (15–18 points) [5]. This study was approved by the Ethics Committee of Peking University Cancer Hospital & Institute, and written informed consent was obtained from all patients.

### Statistics analysis

Mean (standard deviation, SD) or median (interquartile range, IQR) were calculated for continuous variables and frequencies (%) for categorical variables. The Student’s *t*-test or ANOVA is applied for differences between continuous variables and the Pearson chi-square test for categorical variables. Stepwise logistic regression analysis with a forward approach was performed to verify the risk factors of inhospital VTE; results were expressed as odds ratios (OR) with 95% confidence intervals (95% CI). *P*-values < 0.05 (two tailed) were considered as statistically significant. Statistical analysis was performed using the SPSS software package 18.0 (SPSS Inc. USA). The area under the curve (AUC) of the ICU-VTE scores according to the receiver operating characteristic (ROC) curve was performed by the MedCalc® statistical software.

## Results

### Baseline characteristics, tumor-specific factors, treatments, and outcomes

**Fig. 1:**
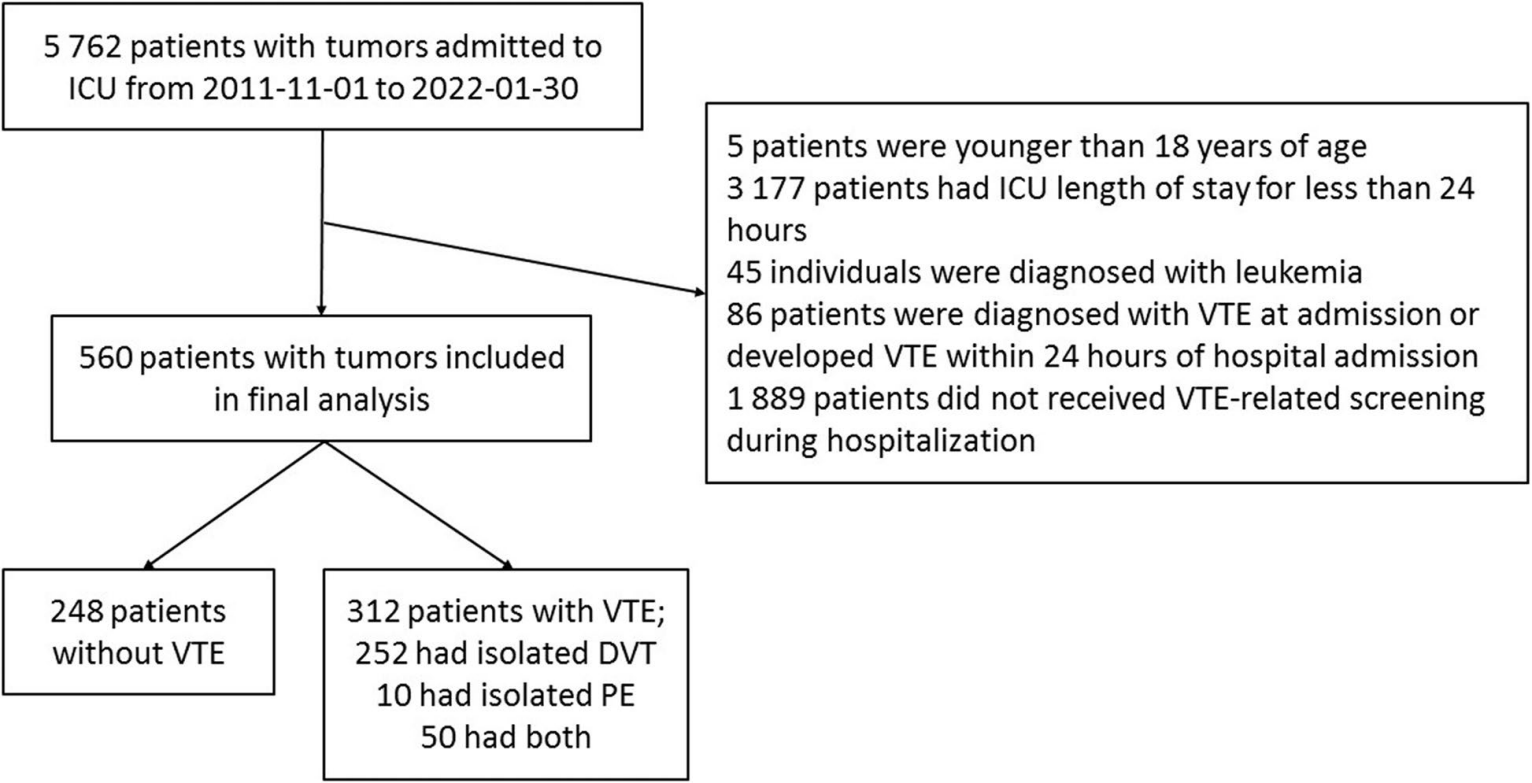
Flowchart showing the final study cohort

Patients’ demographic and clinical characteristics at baseline were listed in Table 1. There were 560 individuals (including 365 males, 65.2%) finally chosen for this study (Fig. 1), with a median age of 65.0 (*IQR* 58.0–72.0) years and a mean BMI of 23.9 (± 3.9) kg/m^2^. The main cause of ICU admission was surgical (397 patients, 70.9%), and medical diseases, such as infection, allergic shock, respiratory failure, or cardiac attack, formed the rest part (163 patients, 29.1%). The median LOS was 21.0 days (range of 2.0–225.0), while ICU LOS had the same range with immobilization duration (4.0 days, range of 1.0–130.0), and the median time for the duration of mechanical ventilation was 1.0 day (range of 1.0–65.0). Blood type A was the most common blood group in the sample (30.8%), followed by B (29.9%), AB (11.3%), and O (28.1%). Gastrointestinal cancer was the most common tumor diagnosis (366 individuals, 65.4%), followed by hepatobiliary and pancreatic tumors (43 individuals, 7.7%) and retroperitoneal masses (40 individuals, 7.1%) (not shown in table). One-hundred sixty-four patients (30.1%) had high-stage tumors (S4), and 227 (41.7%) patients had high-grade tumors (G3–4). Inhospital VTE occurred in 312 patients (55.7%); 252 patients (45.0%) had isolated DVT, 10 patients (1.8%) developed isolated PE, and 50 patients (8.9%) developed both DVT and PE.

**Table 1:** Baseline characteristics of the study subjects

Characteristics	
Patients, *N*	560
Age, y, IQR	65.0 (58.0–72.0)
Male, *N* (%)	365 (65.2)
BMI, ± SD (kg/m^2^)	23.9 ± 3.9
LOS, d, range	21.0 (2.0–225.0)
ICU length of stay, d, range	4.0 (1.0–130.0)
Immobilization duration, d, range	4.0 (1.0–130.0)
Duration of mechanical ventilation, d, range^a^	1.0 (1.0–65.0)
ABO blood group, *N* (%)^b^	
A	171 (30.8)
B	166 (29.9)
AB	63 (11.3)
O	156 (28.1)
Causes of ICU admission, *N* (%)	
Surgical	397 (70.9)
Medical^c^	163 (29.1)
Inhospital VTE, *N* (%)	
VTE	312 (55.7)
DVT	252 (45.0)
PE	10 (1.8)
Both	50 (8.9)
VTE occurrence time, d, range	8.0 (1.0–67.0)

^a^Nine patients were not treated with invasive mechanic ventilation during hospitalization. ^b^ABO blood group was not available for 4 individuals. ^c^Medical diseases included the following: infection, allergic shock, respiratory failure, cardiac attack, and so on. Data are *n* (% of total available data within each column), mean ± standard deviation (SD), median (interquartile range, IQR), or median (range)

### Predictors of inhospital VTE

In univariate analysis, there were a few notable differences between the “with VTE” group and the “without VTE” group. In Table 2, the VTE group included 199 males (63.8%) with a median age of 66.0 (*IQR*, 60.0–73.0) years, which was significantly higher than patients without VTE (*P* = 0.001). Higher complication rates of inhospital VTE were found in patients with higher BMI and higher D-dimer but resulted in no significant correlation (*P* > 0.05). Patients in intensive care for medical diseases had a higher probability of inhospital VTE than those for surgery (107/163, 65.6% vs. 205/397, 51.6%, *P* = 0.002). Anticoagulant prophylaxis during hospitalization could significantly reduce the incidence of VTE (57.4% vs. 71.0%, *P* = 0.004) while given to 63.4% of the overall study population; 98.3% of them received low-molecular-weight heparin, and 1.7% received warfarin or rivaroxaban in a prophylactic dose. It should be mentioned that 205 patients (36.6%) did not receive anticoagulant prophylaxis due to their high risk of bleeding after surgery or severe myelosuppression after antitumor therapy. Other factors with predictive value for VTE included high-grade tumor (G3–4), prior VTE, higher baseline platelet count, fresh frozen plasma (FFP) transfusion, and longer duration of immobilization (*P* < 0.05, Table 2 and Table 3). Patients who developed VTE also had lower hemoglobin levels during admission, longer duration of mechanical ventilation, longer ICU, and hospital LOS than those who did not (*P* < 0.05).

**Table 2:** Patient characters and tumor-specific factors of the study population

Characteristics	With VTE *N* = 312	Without VTE *N* = 248	*P*
*Patient data*			
Age, y, IQR	66.0 (60.0–73.0)	64.0 (57.0–70.8)	0.001
≥ 65 years, *N* (%)	190 (60.9)	114 (46.0)	< 0.001
Male, *N* (%)	199 (63.8)	166 (66.9)	0.437
BMI, ± SD (kg/m^2^)	24.2 ± 3.8	23.6 ± 4.0	0.077
≥ 25 (kg/m^2^), *N* (%)	119 (38.1)	82 (33.1)	0.214
Smoking history, *N* (%)	119 (38.1)	105 (42.3)	0.314
Past alcohol use, *N* (%)	79 (25.3)	64 (25.8)	0.896
*Comorbidities, N (%)*			
Diabetes	57 (18.3)	55 (22.2)	0.251
Hypertension	135 (43.3)	98 (39.5)	0.371
Prior VTE	56 (17.9)	3 (1.2)	< 0.001
MACCE	55 (17.6)	51 (20.6)	0.378
*Causes of ICU admission*			0.002
Surgical, *N* (%)	205 (65.7)	192 (77.4)	
Medical, *N* (%)	107 (34.3)	56 (22.6)	
*Tumor category, N (%)*			0.244
Lung cancer	17 (5.4)	16 (6.5)	
Hepatobiliary and pancreatic tumors	28 (9.0)	15 (6.0)	
Tumors of digestive system	199 (63.8)	167 (67.3)	
Breast cancer	15 (4.8)	3 (1.2)	
Gynecological tumors	11 (3.5)	10 (4.0)	
Retroperitoneal masses	21 (6.7)	19 (7.7)	
Others^*^	21 (6.7)	18 (7.3)	
*Stage*^*a*^			0.513
1–3, *N* (%)	216 (71.1)	165 (68.5)	
4, *N* (%)	88 (28.9)	76 (31.5)	
*Grade*^*a*^			0.002
1–2, *N* (%)	160 (52.6)	158 (65.6)	
3–4, *N* (%)	144 (47.4)	83 (34.4)	
*Baseline test results*			
WBC, ×10^9^/L, IQR	5.9 (4.6–7.3)	5.7 (4.6–7.6)	0.755
Hemoglobin, g/L, IQR	124.0 (103.3–141.0)	123.5 (101.0–141.8)	0.984
Platelet, ×10^9^/L, IQR	198.0 (153.3–266.8)	181.5 (137.5–251.0)	0.044
Creatinine, μmol/L, IQR	64.0 (53.0–74.0)	67.0 (55.0–81.0)	0.742
Albumin, g/L, IQR	41.4 (37.9–44.7)	41.9 (37.4–44.8)	0.479
Bilirubin, μmol/L, IQR	12.6 (9.1–17.6)	12.1 (8.4–16.7)	0.120
Glucose, mmol/L, IQR	5.7 (5.1–6.9)	5.6 (5.0–6.8)	0.985
TC, mmol/L, IQR^b^	4.37 (3.81–5.17)	4.26 (3.52–4.98)	0.039
TG, mmol/L, IQR^b^	1.18 (0.85–1.74)	1.24 (0.85–1.85)	0.299
ABO blood group, *N* (%)^c^			0.216
A	86 (27.8)	85 (34.4)	
B	100 (32.4)	66 (26.7)	
AB	32 (10.4)	31 (12.6)	
O	91 (29.4)	65 (26.3)	
APTT, s, IQR	30.1 (27.6–34.0)	31.0 (28.5–35.3)	0.362
INR, IQR	1.02 (0.97–1.08)	1.04 (0.98–1.14)	0.131
D-dimer, μmol/L, IQR^d^	1.18 (0.75–2.51)	1.03 (0.67–2.12)	0.052
APECHE II	10 (7–13)	9 (7–12)	0.145

^*^Other tumor types included the following: thyroid cancer, prostatic cancer, malignant melanoma, lymphoma, and benign tumor. ^a^Benign lesion was occurred in 15 patients. ^b^Baseline TC and TG examination were not available for 19 individuals. ^c^ABO blood group was not available for 4 individuals. ^d^One-hundred seventy-five patients did not receive D-dimer examination at admission. Data are *n* (% of total available data within each column), mean ± SD, or median (IQR)

**Table 3:** Patient treatments and outcomes

Characteristics	With VTE *N* = 312	Without VTE *N* = 248	*P*
*Treatments*			
CVC, *N* (%)	293 (93.9)	230 (92.7)	0.580
Invasive mechanical ventilation, *N* (%)	307 (98.4)	244 (98.4)	0.992
Transfusion of blood component, *N* (%)	175 (56.1)	122 (49.2)	0104
RBC transfusion, *N* (%)	135 (43.3)	95 (38.3)	0.239
Platelet transfusion, *N* (%)	30 (9.6)	31 (12.5)	0276
Fresh frozen plasma transfusion, *N* (%)	162 (51.9)	105 (42.3)	0.024
TPN, *N* (%)	213 (68.3)	162 (65.3)	0.461
Pharmacologic prophylaxis, *N* (%)	179 (57.4)	176 (71.0)	0.001
*Outcomes*			
Lowest Hb level in hospital, g/dL, IQR	79.5 (69.0–95.0)	82.0 (70.8–101.0)	0.029
Duration of mechanical ventilation, d, range	1 (1–17)	1 (1–8)	0.015
ICU length of stay, d, IQR	4 (2–8)	3 (1–6)	< 0.001
Immobilization duration, d, IQR	4 (2–8)	3 (1–5)	< 0.001
Immobilization ≥ 4 days, *N* (%)	195 (62.5)	89 (35.9)	< 0.001
Hospital length of stay, d, IQR	23.5 (15–36.8)	19 (14–30)	0.005
*The ICU-VTE score, IQR*	11.0 (9.0–12.0)	9.0 (7.0–11.0)	< 0.001
Low risk, 0–8 points, *N* (%)	44 (14.1)	87 (35.1)	< 0.001
Intermediate risk, 9–14 points, *N* (%)	251 (80.4)	161 (64.9)	
High risk, 15–18 points, *N* (%)	17 (5.4)	0	

Data are *N* (% of total available data within each column), mean ± SD, or median (IQR)

Our results were largely consistent with prior study of Viarasilpa T.; therefore, we used the ICU-VTE score to predict the risk of inhospital VTE, the VTE group had notably higher scores (median 11.0, *IQR*, 9.0–12.0 vs. median 9.0, *IQR*, 7.0–11.0, *P* < 0.001, shown in Table 3), and the difference remained significant after divided into three groups (low risk vs. intermediate risk vs. high risk, 33.6% vs. 60.9% vs. 100%, *P* < 0.001).

After the multivariate logistic regression analysis (Table 4), the medium-high-risk group according to the ICU-VTE score was proved to be an effective predictor of inhospital VTE when compared with the low-risk group (9–18 points vs. 0–8 points, *OR*, 3.13; 95% *CI*, 2.01–4.85, *P* < 0.001). We also got some other predictive factors: age ≥ 65 years (*OR*, 1.85; 95% *CI*, 1.28–2.67, *P* = 0.001), high tumor grade (G3–4, *OR*, 1.80; 95% *CI*, 1.24–2.62, *P* = 0.002), medical diseases (*OR*, 1.56; 95% *CI*, 1.03–2.36, *P* = 0.037), FFP transfusion (*OR*, 1.63; 95% *CI*, 1.13–2.37, *P* = 0.010), and anticoagulant prophylaxis (*OR*, 0.55; 95% *CI*, 0.37–0.81, *P* = 0.002). No significant relationship was detected between total cholesterol (TC) and VTE after adjustment.

**Table 4:** Multivariate logistic regression analyses for VTE

Variable	Logistic regression	
	OR (95% *CI*)	*P*
The ICU-VTE score		
9–18 points vs. 0–8 points	3.13 (2.01–4.85)	< 0.001
Age ≥ 65 years	1.85 (1.28–2.67)	0.001
Pathological grade 3–4 vs. grade 1–2	1.80 (1.24–2.62)	0.002
Medical diseases	1.56 (1.03–2.36)	0.037
Fresh frozen plasma transfusion	1.63 (1.13–2.37)	0.010
Pharmacologic prophylaxis	0.55 (0.37–0.81)	0.002

### ROC curves analysis of the ICU-VTE score predictive value for inhospital VTE

**Fig. 2:**
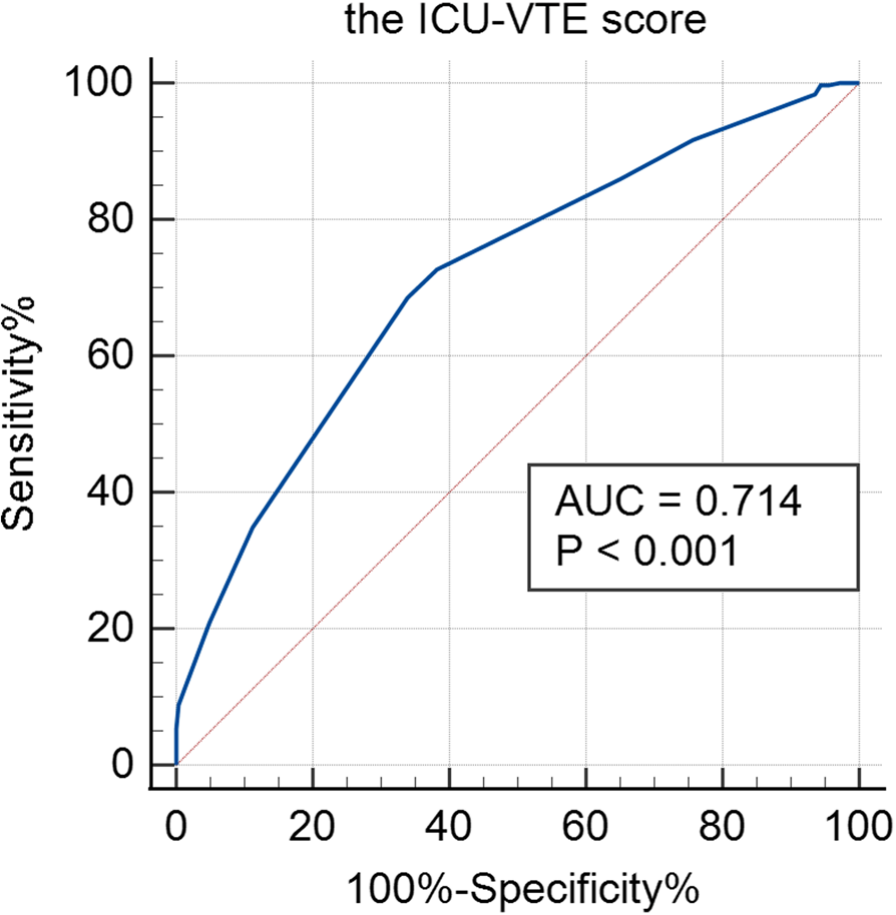
ROC curves analysis of the ICU-VTE score predictive value for inhospital VTE

The AUC of the ICU-VTE scores according to the ROC curve was 0.714 (95% *CI*, 0.67–0.75, *P* < 0.001) (Fig. 2). If the cutoff ICU-VTE score was 10, the sensitivity was 0.686 (95% *CI*, 0.63–0.74), and the specificity was 0.661 (95% *CI*, 0.60–0.72).

## Discussion

VTE is closely related to the length of hospital stay and prognosis and has become the second leading cause of death in tumor patients. ICU patients are considered to be at high risk for VTE even after routine prophylactic anticoagulant therapy (upper and lower extremity venous thrombosis, about 10%) [16, 17]. In recent years, numerous studies have evaluated the risk factors related to VTE from the aspects of patient factors, tumor factors, and treatment factors [3–10, 12, 13], thus developing the thrombosis evaluation scale and model for outpatient and inpatient patients to predict the risk of thrombosis [14, 15]. Recently, an ICU-VTE scale was created for ICU inpatients to assess the risk of thrombosis in critically ill patients [5]. Until now, there has been no validated VTE risk assessment tool that can be applied to medical and surgical critically ill patients with tumors.

This retrospective study evaluated the risk of thrombosis in 560 ICU patients during hospitalization from the aspects of patient factors, tumor factors, and treatment factors, as well as using the ICU VTE scale, and found that multiple factors were closely associated with thrombosis in critical patients with tumors. A total of 63.4% (355 individuals) of our study population with low bleeding risk received anticoagulant prophylaxis, and the inhospital VTE occurrence rate in our cohort was 55.7%, while lower VTE frequency (about 10%) was obtained in previous researches [16–18]. The median interval from ICU admission to VTE diagnosis was 8 days, and most events occurred within the first 2 weeks of ICU admission, which is consistent with previous studies [5, 18].

### Patient characters

Patient factors that increase the risk of VTE include female sex, previous VTE history, advanced age, obesity, and ABO blood type [5–8, 10].

A study of 44,656 patients undergoing surgery for solid tumors elucidated other risk factors of VTE after cancer surgery with a 1.6% overall risk of VTE, such as tumor type, metastatic disease, congestive heart failure, ascites, thrombocytosis, hypoproteinemia, and operation duration > 2 h [19]. Other researchers found that baseline analysis of high-density lipoprotein cholesterol levels might be of clinical value in predicting VTE in cancer outpatients treated with anticancer drugs [20]. We got the same conclusion that age ≥ 65 years, prior VTE, and higher PLT counts contributed to VTE occurrence, while higher TC levels tend to be a novel VTE risk factor but turned to no statistic difference after adjustment. Another study of 43,808 patients undergoing cancer surgery confirmed the effect of coexisting disease on the risk of postoperative VTE; they found that longer hospital stays (> 1 week) and postoperative complications (wound infection, re-intubation, cardiac arrest, and sepsis) were more likely to lead to VTE [21]. This study explains our results laterally that patients admitted to ICU for acute medical problems had a 1.56 times higher risk of VTE than postoperative patients (*OR*, 1.56; 95% *CI*, 1.03–2.36, *P* = 0.037).

We also found that the length of ICU stay and hospital stay in tumor patients treated for acute medical diseases was significantly higher than those in the surgery group (ICU duration, median 8.0 days, *IQR*, 4.0–14.0 days vs. median 3.0 days, *IQR*, 1.0–5.0 days, *P* < 0.001; LOS, median 22.0 days, *IQR*, 16.0–34.0 days vs. median 20.0 days, *IQR*, 13.0–33.0 days, *P* = 0.02; not shown in table), which partly explains the higher incidence of VTE in this group. We thought there was a process of mutually affecting, promoting, and positive interaction.

The link between Hb and VTE remained contradictory results in previous studies [5, 22]; Chi G. confirmed that anemia was independently associated with higher VTE incidence among acutely ill medical patients despite the provision of thromboprophylaxis in an APEX trial substudy including 7513 hospitalized medical patients [23]. We also found the similar trend that patients developed VTE had lower Hb levels during admission (median 79.5 g/L, *IQR*, 69.0–95.0 g/L vs. median 82.0 g/L, *IQR*, 70.8–101.0 g/L, *P* = 0.029). One possible explanation is that anemia may contribute to endothelial dysfunction, blood stasis, and/ or hypercoagulable state, which in turn lead to a greater risk of VTE [24, 25]. Anemia, on the other hand, is often indicative of a number of conditions that can lead to VTE, such as inflammation, surgery, malnutrition, and bone marrow suppression after chemotherapy [1, 8, 11, 21]. Thus, our study offered supporting evidence for hemoglobin measurement as a wildly available and useful method of VTE risk assessment.

### Tumor-specific factors

In recent years, a number of studies have shown that tumor type, stage, and histopathological grade were closely related to VTE [1, 7, 8, 11, 12]. Tumor cells may express the procoagulant activity and induce thrombin production, while noncancerous tissues of patients may also express the procoagulant activity under the influence of tumors [26]. Blood-derived tissue factors in microparticles may play a role in the pathogenesis of hypercoagulability associated with cancer [27]. Some tumors increase the risk of VTE either through external compression or direct invasion of large vessels [28]. One study included 8 million patients older than 65 years who were hospitalized in the USA between 1988 and 1990 and found that patients with a diagnosis of malignancy had a higher incidence of VTE during initial hospitalization, and the malignancies with the highest incidence of VTE included ovarian, brain, pancreatic, and lymphoma [2]. Another large cohort study with 57,591 patients hospitalized for cancer indicated that high tumor stage was associated with increased risk of VTE (incidence rate, 27.7, 95% *CI*, 24.0–32.0) [29]. In addition, the CATS study included 740 patients with solid tumors confirmed high tumor grade (G3–4) to be a significant risk factor of VTE (hazard ratio, 2.0, 95% *CI*, 1.1–3.5) [12]. In this study, we did not find significant differences in the incidence of VTE among patients with different tumor types and stages, while histological grade was proved to be a risk factor of VTE (G3–4 vs. G1–2, 63.4% vs. 50.3%, *OR*, 1.80; 95% *CI*, 1.24–2.62, *P* = 0.002).

### Treatment factors

It has been found that thromboprophylaxis can reduce the risk of VTE in inpatients of internal medicine and surgery [30], and another resent study showed that continuing aspirin can also protect patients with high thromboembolic risk from VTE without increasing bleeding complications during the perioperative period [31]. Meanwhile, Ohta H. reported that fondaparinux administration appeared to be risk factors for postoperative bleeding in patients after colorectal cancer surgery [32]. Therefore, it is necessary to evaluate the bleeding risk and benefits before perioperative antithrombotic therapy carefully. The preferred method for VTE prevention is primary prophylaxis, which include mechanical methods (IPC and GCS) and drugs (low-dose unfractionated heparin, LMWH, fondaparinux, oral factor Xa, or direct thrombin inhibitors) [13, 33]. In this study, all patients admitted to ICU received physical prophylaxis (IPC or GCS); 63.4% of the cohort population with low bleeding risk received drug prophylaxis during hospitalization, of which 349 patients (98.3%) received the recommended dose of LMWH anticoagulant; and 6 patients (1.7%) received oral prophylaxis such as rivaroxaban or dabigatran. We concluded that drug prophylaxis significantly reduced the incidence of VTE in ICU inpatients (*OR*, 0.55; 95% *CI*, 0.37–0.81, *P* = 0.002), validly confirming previous studies.

Invasive mechanical ventilation was proved to be a significant risk factor of VTE because of activity limitation and reduced venous return from positive airway pressure [5, 34], but no significant difference was found in this study. It is worth mentioning that only 9 individuals did not receive ventilation treatment during their hospitalization; the difference would be meaningful if we get a larger sample size. Meanwhile, we got the same conclusion with prior studies that a longer duration of mechanical ventilation, as well as prolonged immobilization and longer hospitalization, resulted in higher VTE occurrence [5].

Several prior researches confirmed that CVC increased VTE incidence by local vessels injury and blood flow stasis [5, 34, 35]; 293 (56.0%) patients with CVC in our study developed VTE, slightly higher than those without CVC (19, 51.4%), but no visible correlation was found.

Blood transfusion was wildly used in tumor operation and myelosuppression after chemotherapy as an alternative treatment in cancer patients [36–39]; both red blood cell (RBC) and platelet transfusions were identified to be predictive variables of VTE (RBC: *OR*, 1.60; 95% *CI*, 1.53–1.67; platelets: 1.20; 1.11–1.29; *P* < 0.001) and inhospital mortality (RBCs: *OR*, 1.34; 95% *CI*, 1.29–1.38; platelet: 2.40; 2.27–2.52; *P* < 0.001) in a retrospective cohort study with 504,208 hospitalizations of patients with cancer between 1995 and 2003 at 60 US medical centers [40]. Several possible mechanisms might be related to this phenomenon: transfusion can improve blood stasis by increasing the circulating red cell mass, severe shortage of nitric oxide in stored red cells might cause vasoconstriction in turn leading to vascular rheologic changes and rising risk of thrombosis, and plentiful pro-inflammatory and pro-thrombotic-soluble mediators such as sCD40L, platelet microparticles, and activated platelets are contained in blood conduct and could contribute to the prothrombotic state in cancer patients [4, 41–43]. In our cohort, patients treated with blood transfusion got higher VTE occurrence (56.1% vs. 49.2%); a significant difference was found in patients who received FFP transfusion after multivariate analysis (*OR*, 1.63; 95% *CI*, 1.13–2.37, *P* = 0.010). Few previous researches reported the relationship between VTE and plasma transfusion; further studies are needed to confirm this association.

### The ICU-VTE scores

At last, we quoted the ICU-VTE score as a new VTE risk assessment model for ICU patients with tumors, which included six proven independent predictors (chosen from patient characters and treatment factors), and results verified the feasibility of this model. Firstly, we found that individuals developed VTE got significantly higher scores than the others (median 11.0, *IQR*, 9.0–12.0 vs. median 9.0, *IQR*, 7.0–11.0, *P* < 0.001) when examining the observed VTE rates across the full range of ICU-VTE scores from 0 to 18, which was consistent with prior research [5]. Secondly, when grouping by scores, low-risk patients (131, 23.4% of the total cohort) with scores of 0–8 have an overall 33.6% rate of VTE, and intermediate-risk patients (412, 73.6% of the study cohort) with scores of 9–14 have an overall 60.9% rate of VTE, while all members of high-risk group (17, 3.0% of the study cohort) with 15–18 scores experienced inhospital VTE, and the rate was 1.8 times the risk of VTE among all patients. Thirdly, tumor patients of intermediate- and high-risk group with 9–18 scores had statistically significant higher rates of VTE after adjustment (62.5% vs. 33.6%, *OR*, 3.13; 95% *CI*, 2.01–4.85, *P* < 0.001). At last, our analysis of ROC curves showed that an ICU-VTE score of > 10 was a significant predictor of inhospital VTE, almost consistent with the cutoff ICU-VTE score presented by Viarasilpa T.

The relatively higher VTE rates, when compared with prior studies, might be related to disease feature (all patients were diagnosed with tumor), ethnicity, and the majority of our study population were treated with surgery (523, 93.4%), CVC (523, 93.4%), and invasive mechanical ventilation (551, 98.4%) in hospital, which were proven to be independent risk factors of VTE in prior studies [5, 19, 34, 35]. Moreover, due to it being a retrospective study, not all of the patients hospitalized in ICU received VTE-related screening, and data on VTE events after hospitalization were unable to be obtain, which lead to relatively small sample size, incomplete information, and skewed distribution of study population. Finally, other potential effects like chemotherapy was not included in this study. These limitations may cause the obtained results correspondingly short of conviction.

## Conclusions

We got the conclusion that the ICU-VTE score was independently associated with inhospital VTE risk in ICU patients with tumors, as well as age ≥ 65 years, plasma transfusion, high tumor grade (G3–4), and hospitalized for medical diseases, while pharmacologic prophylaxis during admission was proved to be protective against VTE. In addition to those mixed critically ill patients, we believe that the ICU-VTE score can also provide accurate inhospital VTE risk stratification among ICU patients with tumors. The predictive accuracy might be improved when combined with tumor-specific factors such as histologic grade; therefore, further follow-up researches are needed to confirm it.

## Supplementary Information

**Additional file 1.** VTE.

## Data Availability

The data will be shared after publishing.

## Abbreviations

ICU: Intensive care unit
VTE: Venous thromboembolism
DVT: Deep venous thrombosis
PE: Pulmonary embolism
TPN: Total parenteral nutrition
CTPA: CT pulmonary angiography
MACCE: Main adverse cardiovascular and cerebrovascular events
PT: Prothrombin time
INR: International normalized ratio
APTT: Activated partial thromboplastin time
CVC: Central venous catheterization
GCS: Graduated compression stocking
IPC: Intermittent pneumatic compression
V/Q: Ventilation-perfusion
LOS: Length of stay
SD: Standard deviation
IQR: Interquartile range
OR: Odds ratio
CI: Confidence interval
AUC: Area under the curve
ROC: Receiver operator characteristic
TC: Total cholesterol
TG: Triglycerides
WBC: White blood cell
RBC: Red blood cell
BMI: Body mass index
FFP: Fresh frozen plasma
APECHE: Acute physiology and chronic health evaluation
LMWH: Low-molecular-weight heparin
